# Collagen-V and K-α-1 Tubulin Antibodies as Potential Markers of Unsuspected GERD-Related Lung Damage: Insights from a Cross-Sectional Analysis

**DOI:** 10.1007/s00408-024-00745-8

**Published:** 2024-09-24

**Authors:** Andrés R. Latorre-Rodríguez, Sumeet K. Mittal, Ranjithkumar Ravichandran, Mark Shacker, Andrés Isaza-Restrepo, Sandhya Bansal, Thalachallour Mohankumar, Ross M. Bremner

**Affiliations:** 1 Norton Thoracic Institute, St. Joseph´s Hospital and Medical Center, Phoenix, AZ USA; 2 Grupo de Investigación Clínica, Escuela de Medicina y Ciencias de la Salud, Universidad del Rosario, Bogotá, Colombia; 3 School of Medicine, Creighton University, Phoenix, AZ USA

**Keywords:** Gastroesophageal reflux, Hiatal hernia, Respiratory aspiration, Lung injury, Lung fibrosis, Extracellular vesicles, Biomarkers

## Abstract

**Purpose:**

Our group has proposed that aspiration of gastric contents leads to exposure of normally sequestered lung self-antigens (SAgs), specifically collagen-V (Col-V) and K-α-1-tubulin (Kα1T), which elicits an immune response characterized by increasing concentrations of self-antibodies (SAbs) anti-Col-V and anti-Kα1T. We sought to establish the point prevalence of abnormally elevated concentrations of SAbs among patients with pathological gastroesophageal reflux disease (GERD) and/or hiatal hernia undergoing antireflux surgery (ARS).

**Methods:**

For this cross-sectional study, we retrieved a plasma aliquot from the Norton Thoracic Institute BioBank from blood samples that were taken preoperatively from patients who underwent ARS between November 2019 and August 2022. Enzyme-linked immunosorbent assays were employed to detect and quantify anti-Col-V and anti-Kα1T.

**Results:**

Samples from 43 patients (females, *n* = 34 [79.1%]; mean age, 62 ± 12 years; and mean BMI, 30.5 ± 7  kg/m^2^) were analyzed. Before ARS, 28 (65.1%, CI95: 50.3–80.0%) patients had abnormally elevated concentrations of anti-Col-V and 19 (44.2%, CI95: 28.7–59.7%) had elevated concentrations of circulating anti-Kα1T. Overall, 13 patients (30.2%) had low (i.e., normal) concentrations of both SAbs, 13 (30.2%) were positive only for one, and 17 (39.5%) were positive for both SAbs.

**Conclusion:**

A relative high point prevalence of abnormally elevated circulating SAbs (i.e., anti-Col-V and/or anti-Kα1T) before ARS was found. This result suggests clinically unsuspected pulmonary parenchymal injury secondary to GERD-related aspiration. Further studies are required to confirm this hypothesis and to identify alternative non-invasive early biomarkers of GERD-related lung damage.

**Supplementary Information:**

The online version contains supplementary material available at 10.1007/s00408-024-00745-8.

## Introduction

Nearly a millennium ago, Rabbi Moses Maimonides advised against overeating and/or lying down immediately after meals as he believed it could lead to cardiopulmonary disease [1]. This is likely the first description of a potential link between reflux and pulmonary disease. Nevertheless, the association between gastroesophageal reflux disease (GERD) and lung disease has been largely disregarded in the medical community since then. Over the last few decades, mainly from the lung transplant community, there has been increasing recognition of the deleterious role that GERD-associated micro- and macro-aspiration plays in lung disease. Unfortunately, this has not been thoroughly explored except in lung transplant patients and patients with fairly advanced stages of pulmonary fibrosis. However, the rapidly increasing worldwide incidence and prevalence of GERD has brought this concern to attention [2].

Multiple pathophysiological mechanisms of how GERD can lead to lung disease have been proposed [3–5]. The two most widely accepted theories are (1) direct mechanical and/or chemical damage (i.e., epithelial erosion, local damage, and inflammation) caused by exposure of the respiratory epithelium to gastric contents secondary to repetitive micro- and macro-aspiration and (2) activation of neural pathways, including vagal reflexes, which may explain symptoms like bronchoconstriction and chronic cough [3, 4]. However, our previous work in transplant patients and a murine aspiration model has shown that aspiration-induced pulmonary damage results in an autoimmune response to normally sequestered lung self-antigens (SAgs) which potentiates pulmonary injury [6–10].

The proposed pathophysiological mechanisms imply that aspiration of gastric contents causes direct mechanical and/or chemical damage to the respiratory epithelium. Then, SAgs, specifically collagen-V (Col-V) and K-α-1 tubulin (Kα1T), are exposed and recognized by the immune system, which targets them. This results in the expression of self-antibodies (SAbs) against Col-V and Kα1T, which can be measured in peripheral blood [7, 8, 11].

Therefore, we hypothesize that measuring this immune response (i.e., SAgs/SAbs) among patients with GERD may help identify patients with clinically unsuspected lung damage, which ultimately could allow early, patient-tailored therapy. As a first step, we sought to determine the point prevalence of abnormally elevated concentrations (i.e., seropositivity) of SAbs found in peripheral blood samples taken from patients before antireflux surgery (ARS).

## Methods

### Study Design and Ethics

This retrospective, cross-sectional analysis reports the preoperative seropositivity for anti-Col-V and anti-Kα1T among patients who underwent ARS at a tertiary surgical center in the United States. Plasma samples from patients who consented to participate in an institutional bio-banking protocol were retrieved and analyzed. Relevant clinical data extraction from patient charts and publication of results were approved by the Institutional Review Board of St. Joseph’s Hospital and Medical Center, Phoenix, AZ, under the Norton Thoracic Institute Biorepository Protocol (OSRA# 18-500-251-73-18, bio-banking approval: date: 23-Feb-2019, project approval: 15-May-2023). Written patient consent for bio-banking was verified; for this report, an additional consent was waived due to its design. All research activities were conducted following good practice guidelines according to the 2013 Helsinki Declaration. The STrengthening the Reporting of OBservational studies in Epidemiology (STROBE) statement and checklist were followed to guide the reporting of results (Online Resource 1).

### Study Population

Consecutive patients presenting with a diagnosis of GERD and/or hiatal hernia (HH) who underwent ARS (i.e., including HH repair with fundoplication or non-bariatric Roux-en-Y reconstruction) between November 2019 and August 2022 and had available stored preoperative plasma samples (i.e., > 500 µL) at the institutional biobank were included in the study. Patients who were < 18 years old, underwent other surgical procedures, had a history of foregut malignancies (e.g., gastric or esophageal adenocarcinoma) or lung transplant, or did not have adequate volumes of stored plasma were excluded from analysis.

### Outcomes

The *primary outcome* was the preoperative prevalence of seropositivity of anti-Col-V and anti-Kα1T among patients with GERD/HH prior to ARS. The *secondary outcome* was the preoperative seropositivity of SAbs according to the primary surgical indication.

### Preoperative Workup

All patients underwent an in-person appointment with an expert foregut surgeon before ARS, during which a medical history was taken and a physical evaluation was performed. Surgery is offered to patients after a thorough workup, including esophagogastroduodenoscopy (EGD), barium esophagogram, high-resolution manometry (HRM), and pH monitoring (selectively).

Briefly, available preoperative EGD reports were extracted, and data on objective evidence of GERD (i.e., erosive esophagitis [Los Angeles grade ≥ B], Barrett’s esophagus, and peptic strictures) as well as the presence of HH was collected. HRM studies were conducted using a solid-state system catheter with 36 channels at 1-cm intervals (Medtronic, Minneapolis, MN), and all studies were re-analyzed by an experienced interpreter (AL) following the Chicago Classification 4.0 diagnostic criteria. When indicated, pH monitoring was completed using conventional ambulatory 24-hour pH monitoring with a transnasal catheter system (Digitrapper, Medtronic, Minneapolis, MN). Either a DeMeester score ≥ 14.73 or a total acid exposure time > 6% was considered positive for pathological reflux. Finally, two dichotomous variables (i.e., esophageal-esophageal reflux and gastroesophageal reflux) were extracted from the radiological reports of barium esophagrams.

### Blood Sampling and Bio-Banking

Blood samples of patients enrolled in the biorepository protocol are taken immediately before the surgical procedure using tubes containing ethylenediaminetetraacetic acid (EDTA). Thereafter, the blood is centrifuged at 1200 × g for 10 min, and the isolated plasma is stored in aliquots of 500  µL at −80  °C. For this study, one aliquot was retrieved from each patient for further analysis.

### Assessment of Serum SAb Concentrations

Enzyme-linked immunosorbent assays (ELISA) were conducted to quantify concentrations of antibodies against Col-V and Kα1T as described elsewhere [7]. Briefly, ELISA plates were coated overnight at 4  °C with either Col-V (1  µg/mL; Sigma-Aldrich, St. Louis, Missouri, USA) or recombinant Kα1T (1  µg/mL) in phosphate-buffered saline, then blocked for 2 h with 1% bovine serum albumin. Samples were diluted to 1:1000 for Col-V and 1:1250 for Kα1T and then incubated overnight at 4 °C. Thereafter, using tetramethylbenzidine substrate, a colorimetric reaction was developed, and specific SAbs were detected using horseradish peroxidase-conjugated anti-human IgG (1:10,000). Samples were read at 450  nm. Seropositivity was considered at concentrations ≥ 106  ng/mL for anti-Col-V and ≥ 116  ng/mL for anti-Kα1T (the cut-off is based on the mean ± 2 standard deviations for samples of historical healthy controls) [7, 8]. Concentrations were calculated using standard curves from known concentrations of anti-Col-V (Abcam, Cambridge, United Kingdom) and anti-Kα1T (Santa Cruz Biotechnology, Dallas, TX). All antibody analyses were performed in triplicate.

### Statistical Analysis

Descriptive statistics were used to summarize the demographic and clinical characteristics of the cohort. Median and interquartile range (IQR) or mean and standard deviation (SD) were used to summarize continuous variables, whereas counts and percentages were used for categorical variables. In the case of missing data among categorical covariates, denominators were adjusted as appropriate. Prevalence estimates with 95% confidence intervals (CIs) for anti-Col-V and/or anti-Kα1T seropositivity were computed among the entire cohort and according to the surgical indication. A tabulation summarizing the main preoperative findings and laboratory results of each case was created. Further, differences in terms of SAb concentration and prevalence between groups (i.e., according to primary surgical indication or esophageal body contractility) were assessed using the Chi-square test or the Mann-Whitney U test. All analyses were completed using SPSS v29.0 (IBM SPSS Statistics; Armonk, NY).

## Results

### Demographic and Clinical Characteristics

**Fig. 1:**
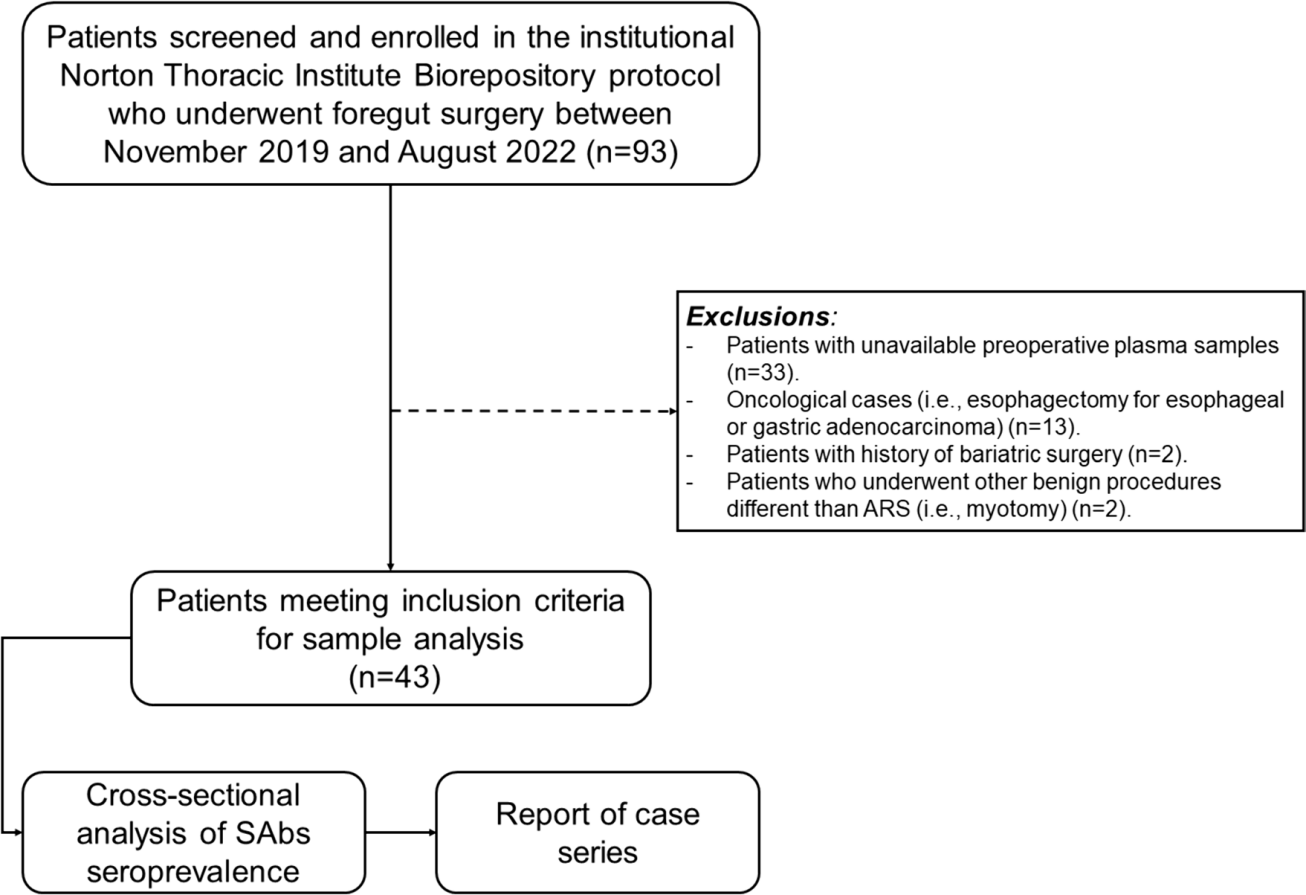
Study flow diagram

Within the study period, 93 patients were enrolled in the bio-banking protocol, and plasma samples from 43 patients who met the inclusion criteria were retrieved. Figure 1 presents the study flow diagram and the reasons for exclusion. Most patients were female (*n* = 34, 79.1%), the median age was 66 years (IQR 53–69), and the median BMI was 29.4  kg/m^2^ (IQR 25.9–33.3). A preoperative American Society of Anesthesiologists grade ≤ II (physical status classification) and a Charlson Comorbidity Index ≤ 3 were reported for 34 (79.1%) individuals.

A remote history of smoking was reported by 19 (44.2%) individuals. In terms of respiratory conditions, 8 (18.6%) patients had a diagnosis of asthma, 6 (14%) of recurrent pneumonia, 6 (14%) of any interstitial lung disorder, 4 (9.3%) of sleep apnea, and 3 (7%) of pulmonary nodules. All patients except one were on acid suppression therapy (*n* = 42, 97.8%), and 41 (95.3%) had a hiatal hernia on endoscopic assessment. The complete demographic and clinical characteristics, medication use, and esophageal testing results are presented in Table 1.

**Table 1:** Summary of preoperative demographic and clinical characteristics of included patients

Covariate	Cohort (*n* = 43)
*Demographics*	
Age, years, median (IQR)	66 (53–69)
Sex, female	34 (79.1)
BMI, kg/m^2^, median (IQR)	29.4 (25.9–33.3)
*Clinical characteristics and physical status*	
Current or former smoker	19 (44.2)
ASA classification ≤ II	34 (79.1)
Charlson comorbidity index ≤ 3	34 (79.1)
History of antireflux surgery	7 (16.2)
*Medication use*	
Acid suppression therapy	42 (97.6)
Antacids	10 (23.3)
H2-blockers	14 (32.6)
Proton pump inhibitors	38 (88.4)
Chronic use of corticoids	1 (2.3)
*History of respiratory disorders*	
Asthma	8 (18.6)
Idiopathic pulmonary fibrosis	6 (14)
Recurrent pneumonia	6 (14)
Obstructive sleep apnea	4 (9.3)
Pulmonary nodules	3 (7)
*Esophageal functional testing*	
Preoperative EGD completed	43 (100)
Endoscopic evidence of GERD	32 (74.4)
Evidence of hiatal hernia	41 (95.3)
Preoperative HRM completed	37 (86)
Normal motility	21 (56.8)
Ineffective esophageal motility	14 (37.8)
EGJ outflow obstruction	2 (5.4)
Preoperative barium esophagram completed	39 (90.7)
Evidence of esophago-esophageal reflux	20 (51.3)
Evidence of gastro-esophageal reflux	21 (53.8)
Preoperative pH monitoring completed	13 (30.2)
DeMeester score, median (IQR)	41.8 (26.8–59.3)
Total AET %, median (IQR)	10.7 (8–17.5)
Upright AET %, median (IQR)	12.5 (10.7–20.3)
Supine AET %, median (IQR)	5.4 (1.8–13.5)
No. of proximal refluxes, median (IQR)	17 (13–27)

All data presented as no. (%) unless otherwise specified. Denominators were adjusted to the completed number of studies as appropriate

*AET* acid exposure time, *ASA* American Society of Anesthesiologists, *BMI* body mass index, *EGD* esophagogastroduodenoscopy, *HRM* high-resolution manometry, *IQR* interquartile range

### Overall Seroprevalence of SAbs Before ARS

**Fig. 2:**
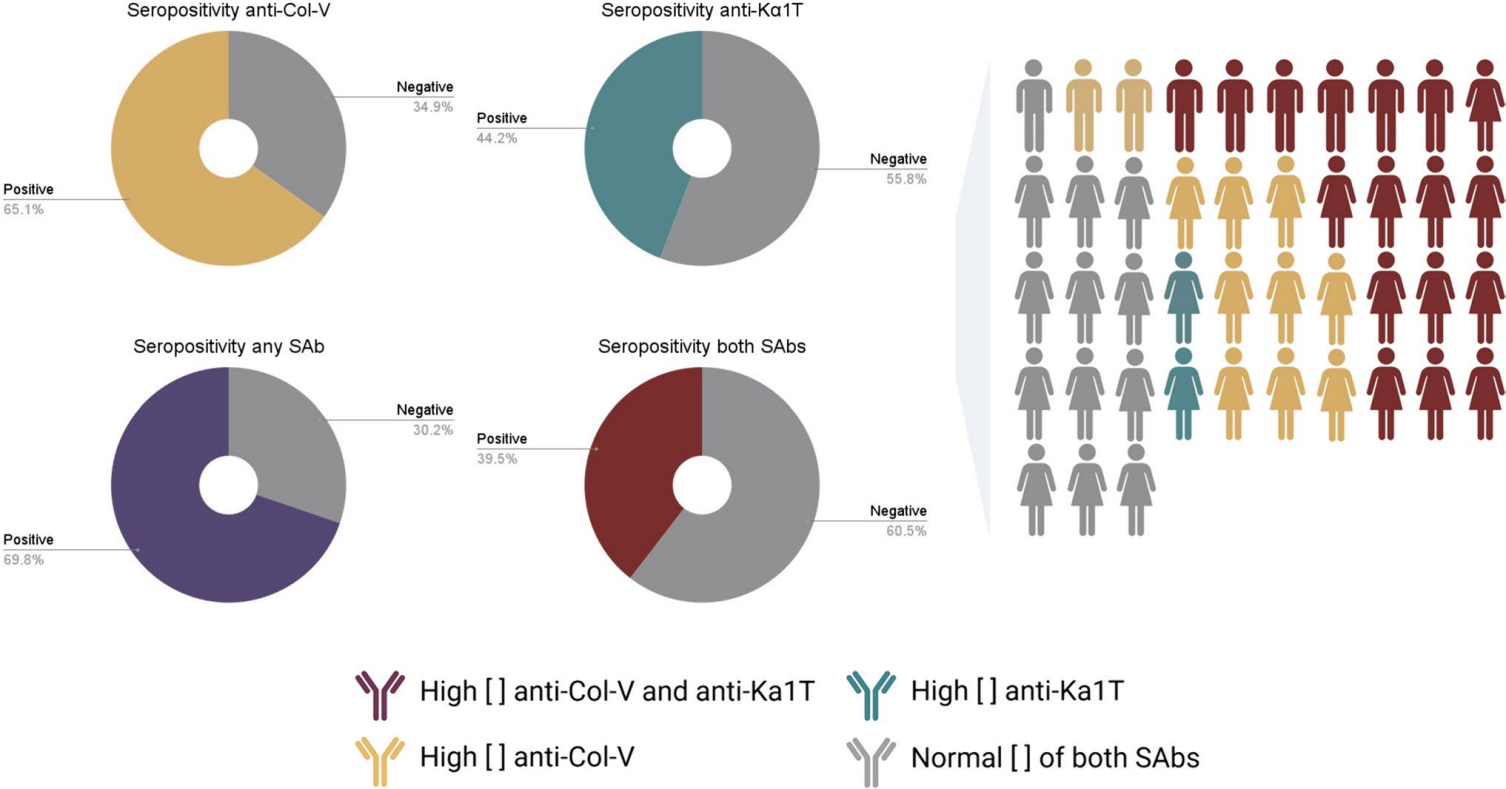
Overall seropositivity of anti-collagen-V (Col-V) and anti-K-α-1 tubulin (Kα1T) among patients who underwent antireflux surgery between November 2019 and August 2022

A comprehensive summary of the demographic and clinical characteristics and preoperative workup of each patient is presented in Table 2. Twenty-eight (65.1%, [95% CI: 50.3–80.0%]) patients had abnormally elevated concentrations of anti-Col-V, and 19 (44.2%, [95% CI: 28.7–59.7%]) had elevated concentrations of circulating anti-Kα1T before ARS. Only 13 (30.2%, [95% CI: 18.5–45.2%]) patients had low (i.e., normal) concentrations of both SAbs, whereas 13 (30.2%, [95% CI: 18.5–45.2%]) were positive for only one antibody, and 17 (39.5%, [95% CI: 26.3–54.4%]) were positive for both SAbs. Figure 2 summarizes the cohort’s overall seropositivity for anti-Col-V and anti-Kα1T.

**Table 2:** Summary of preoperative endoscopic, radiological, and functional esophageal testing as well as the measurements of self-antibodies (SAbs) anti-collagen-V (Col-V) and anti-K-α-1 tubulin (Kα1T) among a group of 43 patients with gastroesophageal reflux disease (GERD) and/or hiatal hernia (HH) undergoing antireflux surgery

No	Age	Sex	BMI, kg/m^2^	CCI	Primary surgical indication	Smoking status	History of respiratory conditions	Evidence of GERD on EGD*	Endoscopic HH	EER/GER on BE	HRM diagnosis	pH-monitoring test	Anti-Col-V status	Anti-Col-V (ng/mL)	Anti-Kα1T status	Anti-Kα1T (ng/mL)
1	69	F	35.6	0	Anemia/bleeding	Never	–	Yes	Yes	EER	IEM	–	Pos (+)	191.48	Pos (+)	169.96
2	52	M	22.1	4	Volume reflux	Former	–	Yes	Yes	GER	IEM	–	Pos (+)	208.77	Pos (+)	289.88
3	77	F	27.8	1	PEH/ITS	Former	–	Yes	Yes	EER, GER	Normal	–	Neg (-)	95.74	Neg (-)	73.33
4	77	F	33.3	3	Anemia/bleeding	Former	–	Yes	Yes	GER	IEM	–	Neg (-)	51.86	Neg (-)	96.63
5	74	F	33.1	0	Anemia/bleeding	Former	–	No	Yes	EER	Normal	–	Neg (-)	102.39	Pos (+)	128.55
6	55	F	25.1	2	Volume reflux	Never	–	Yes	Yes	No	IEM	–	Pos (+)	131.64	Neg (-)	81.96
7	72	F	26.5	4	Obstructive symptoms	Never	Asthma	Yes	Yes	EER	Normal	–	Neg (-)	3.6	Neg (-)	50.04
8	68	F	31.7	0	Volume reflux	Never	OSA	No	Yes	EER	Normal	–	Neg (-)	98.4	Pos (+)	192.39
9	57	F	23.8	3	PEH/ITS	Former	–	No	Yes	EER	Normal	–	Neg (-)	37.23	Neg (-)	33.65
10	37	M	33.3	0	Volume reflux	Former	OSA	Yes	Yes	GER	Normal	Positive	Pos (+)	219.41	Pos (+)	184.63
11	57	F	29.6	4	Volume reflux	Never	–	Yes	Yes	EER	Normal	Positive	Pos (+)	111.7	Neg (-)	94.9
12	41	M	28.2	1	Volume reflux	Never	–	Yes	Yes	No	Normal	Positive	Pos (+)	214.09	Neg (-)	96.63
13	76	F	33.3	1	PEH/ITS	Former	Recurrent pneumonia	No	Yes	GER	Normal	–	Neg (-)	99.73	Neg (-)	83.69
14	60	M	22.8	1	Volume reflux	Former	–	Yes	Yes	EER	IEM	–	Pos (+)	337.75	Pos (+)	270.04
15	53	F	37.8	0	PEH/ITS	Former	–	Yes	Yes	No	IEM	Positive	Neg (-)	101.06	Neg (-)	69.88
16	64	F	28.3	2	Volume reflux	Never	Bronchitis	Yes	Yes	GER	Normal	–	Pos (+)	117.02	Neg (-)	74.2
17	66	F	29.4	5	Volume reflux	Never	IPF	Yes	No	EER	EGJOO	–	Pos (+)	396.26	Pos (+)	174.27
18	48	F	48.6	2	PEH/ITS	Current	Asthma	Yes	Yes	GER	–	–	Neg (-)	61.17	Neg (-)	29.33
19	26	F	32.1	3	Volume reflux	Never	–	Yes	Yes	EER, GER	IEM	Positive	Neg (-)	62.5	Neg (-)	111.29
20	69	F	28.9	0	Volume reflux	Never	Bronchitis, Recurrent pneumonia	Yes	Yes	–	Normal	Positive	Neg (-)	81.11	Neg (-)	113.88
21	67	F	28.8	3	Volume reflux	Never	Asthma	Yes	Yes	EER	IEM	–	Pos (+)	146.27	Neg (-)	104.39
22	69	M	27.6	2	Volume reflux	Former	IPF	Yes	Yes	EER, GER	IEM	Positive	Pos (+)	227.38	Pos (+)	146.67
23	73	F	25.9	3	Volume reflux	Former	IPF, Recurrent pneumonia	Yes	Yes	EER, GER	IEM	–	Pos (+)	1109.93	Pos (+)	224.62
24	69	M	29.4	2	Volume reflux	Former	Recurrent pneumonia, Asthma, OSA	Yes	Yes	EER, GER	IEM	–	Pos (+)	168.88	Neg (-)	97.49
25	44	F	31.5	3	Volume reflux	Former	–	Yes	Yes	No	Normal	Positive	Pos (+)	473.38	Pos (+)	156.16
26	65	F	49.5	4	PEH/ITS	Never	Pulmonary nodules, Asthma	No	Yes	No	IEM	–	Pos (+)	246	Neg (-)	94.01
27	72	F	34.9	1	Obstructive symptoms	Never	–	Yes	Yes	No	Normal	–	Pos (+)	377.79	Neg (-)	59.07
28	66	F	24.6	3	PEH/ITS	Never	–	Yes	Yes	GER	EGJOO	–	Neg (-)	67.36	Neg (-)	79.03
29	71	F	26.3	2	Volume reflux	Never	–	Yes	Yes	GER	Normal	–	Pos (+)	143.5	Neg (-)	83.19
30	63	F	31.5	3	Obstructive symptoms	Never	–	No	Yes	–	Normal	–	Pos (+)	442.21	Pos (+)	178.87
31	71	M	21.5	4	Anemia/bleeding	Former	–	No	Yes	EER, GER	–	–	Pos (+)	656	Pos (+)	155.57
32	46	F	51.7	2	Volume reflux	Never	–	No	Yes	GER	IEM	Positive	Pos (+)	155.58	Pos (+)	128.55
33	69	F	19.6	3	Volume reflux	Never	–	Yes	Yes	GER	–	–	Neg (-)	2.93	Neg (-)	51.58
34	64	F	28.3	2	Volume reflux	Never	Asthma	Yes	No	EER, GER	Normal	Positive	Neg (-)	43.93	Neg (-)	39.93
35	52	M	38.7	6	Obstructive symptoms	Never	IPF, Asthma, Recurrent pneumonia	Yes	Yes	No	Normal	–	Pos (+)	244.67	Pos (+)	154.43
36	56	F	23.4	4	Volume reflux	Never	Pulmonary nodules	Yes	Yes	EER, GER	Normal	–	Pos (+)	1947.5	Pos (+)	529.11
37	64	F	26.3	4	Obstructive symptoms	Never	Pulmonary nodules	Yes	Yes	–	Normal	Positive	Pos (+)	533	Pos (+)	188.02
38	67	F	34.1	2	Volume reflux	Never	–	Yes	Yes	GER	IEM	Positive	Pos (+)	161.07	Pos (+)	130.61
39	68	F	24.6	3	Anemia/bleeding	Never	–	No	Yes	No	–	–	Pos (+)	342.64	Pos (+)	135.61
40	67	M	24.2	3	PEH/ITS	Former	IPF, Recurrent pneumonia	No	Yes	–	Normal	Negative	Neg (-)	46.86	Neg (-)	78.2
41	53	F	31.3	2	Volume reflux	Former	IPF	No	Yes	EER	Normal	–	Pos (+)	184.5	Pos (+)	154.74
42	75	F	35.5	2	Obstructive symptoms	Former	OSA	Yes	Yes	EER, GER	–	–	Pos (+)	480.29	Neg (-)	49.92
43	36	F	32.2	0	Volume reflux	Current	Asthma	Yes	Yes	EER, GER	–	–	Pos (+)	246	Neg (-)	98.17

*BE* barium esophagram, *BMI* body mass index, *CCI* Charlson Comorbidity Index, *EER* esophago-esophageal reflux, *EGD* esophagogastroduodenoscopy, *EGJOO* esophagogastric outflow obstruction, *F* female, *GER* gastroesophageal reflux, *HRM* high-resolution manometry, *IEM* ineffective esophageal motility, *M* male, *IPF* idiopathic pulmonary fibrosis, *ITS* intrathoracic stomach, *Neg (-)* negative, *OSA* obstructive sleep apnea, *PEH* paraesophageal hernia, *Pos (+)* positive

*Objective endoscopic evidence of GERD is defined as the presence of erosive esophagitis (Los Angeles grade ≥ B), Barrett’s esophagus, or peptic strictures

### SAbs According to Surgical Indication

Notably, ARS can be indicated for multiple reasons. Hence, patients were grouped based on the underlying pathophysiological mechanisms and clinical presentations to analyze the prevalence of SAbs according to the primary indication for surgery. Briefly, 24 (55.8%) patients presented with volume reflux (GERD group), and among this subset, 20 (83.3%, [95% CI: 63.5–93.9%]) patients had abnormally elevated concentrations for at least one SAb. The seropositivity prevalence for anti-Col-V and anti-Kα1T was 79.2% (95% CI: 59.1–91.2%) and 50% (95% CI: 31.4–68.6%), respectively. On the other hand, 19 (44.2%) patients underwent ARS primarily because of mechanical concerns (HH group), including obstructive symptoms, intrathoracic stomach, or chronic bleeding. In these cases, seropositivity for at least one SAb trended lower and was noted in only 10 (52.6% [95% CI: 31.7–72.7%]) individuals; moreover, the seropositivity for anti-Col-V was 47.4% (95% CI: 27.3–68.3%) and for anti-Kα1T was 36.8% (95% CI: 19.1–59.1%). In summary, seropositivity for anti-Col-V was significantly higher among in the GERD group than the HH group (79.2% vs. 47.4%, *p* < 0.05), and a trend toward higher seropositivity of anti-Kα1T within the GERD group (50% vs. 36.8%, *p* = 0.39) was also noted (Table 3).

**Table 3:** Prevalence and concentrations of self-antibodies (SAbs) anti-collagen-V (Col-V) and anti-K-α-1 tubulin (Kα1T) among the cohort and according the surgical indication

Covariate	Cohort (*n* = 43)	GERD group (*n* = 24)	HH group (*n* = 19)	*p*-value
SAb seroprevalence				
Seropositivity for any SAb	30 (69.8)	20 (83.3)	10 (52.6)	**0.029**
Seropositivity for anti-Col-V	28 (65.1)	19 (79.2)	9 (47.4)	**0.030**
Anti-Col-V concentration, mean, ± SD	259.8 ± 334.5	291.2 ± 414.7	220.1 ± 196.1	0.616
Seropositivity for anti-Kα1T	19 (44.2)	12 (50)	7 (36.8)	0.388
Anti-Kα1T concentration, mean, ± SD	128.8 ± 86.5	151.2 ± 102.3	100.4 ± 50.5	**0.035**

Bold values denote statistical significance (p < 0.05)

All data presented as no and (%) unless otherwise specified

*GERD* gastroesophageal reflux disease, *HH* hiatal hernia

### SAbs According to Esophageal Peristalsis

A total of 14 (37.8%) of 37 patients with an available HRM study had ineffective esophageal peristalsis (IEM). All patients with IEM had evidence of HH. Although it was not considered a study outcome a priori, we also identified a slight trend (i.e., not statistically significant) toward higher preoperative seropositivity of anti-Col-V (78.6% vs. 56.5%, *p* = 0.17), anti-Kα1T (50% vs. 43.5%, *p* = 0.69), or either SAb (78.6% vs. 65.2%, *p* = 0.39) among patients with a manometric diagnosis of IEM.

## Discussion

The complex relationship between GERD and respiratory disorders remains controversial due to a lack of evidence of clear, conclusive causality, although recent studies support this relationship [12]. There is an increasing body of evidence supporting the role of GERD in facilitating lung damage and, similarly, in a bi-directional fashion, the role of pulmonary disorders in the development of GERD [11, 13–15]. Furthermore, the Pulmonary Fibrosis Consensus Group of the American Thoracic Society, European Respiratory Society, Japanese Respiratory Society, and Asociación Latinoamericana de Tórax extensively debate the role of GERD in the development and progression of idiopathic pulmonary fibrosis (IPF) in recently updated clinical practice guidelines [16]; on the one hand, the development of IPF is multifactorial, but on the other hand, the role of GERD as either a trigger or contributor to other underlying causes cannot be ignored.

Further, the deliberated chronic use of acid suppression therapy may accentuate the consequences of repetitive silent micro-aspiration. As documented in our study, almost all GERD patients (97.2%) received acid suppression therapy before mechanical control (i.e., ARS); however, although these medications increase the pH of refluxed material and control the most frequent symptoms, their use does not prevent aspiration events. Of note, nearly all studies looking for a treatment for GERD in IPF patients have incorporated and evaluated the use of acid suppression therapy, and their results are conflicting [17]. While some favor the use of proton pump inhibitors (providing stabilization of functional lung capacity and reduction of disease exacerbations), others report that their use in patients with IPF do not provide additional benefits and even leads to unexpected complications (e.g., increased risk of respiratory infections [17].

On the other hand, case series and studies have reported that mechanical control of reflux (i.e., ARS) is safe and improves pulmonary function; however, the inference is very limited due to the nature of this observation [18–20]. In the randomized controlled trial WRAP-IPF by Raghu et al. [21], the authors explored the protective role of ARS in patients with IPF. They found a trend toward fewer respiratory-related complications and lower mortality among patients who underwent ARS compared to those who were medically treated. Unfortunately, the study was limited not only by the sample size but also, more importantly, by the inclusion of only patients with late-stage IPF [21].

In our opinion, the resulting quagmire is caused by the absence of early non-invasive and reliable biomarkers of GERD-related pulmonary disease, which often goes clinically unsuspected and is managed symptomatically until late stages. A reliable non-invasive marker of pulmonary parenchymal injury would greatly improve diagnosis and hence treatment in the early stages of the disease. If such a marker is associated with certain phenotypes of GERD, developing strategies for the definitive treatment of GERD will improve patient management and potentially lead to the prevention of continued lung injury.

The expected prevalence of seropositivity of antibodies against normally sequestered SAgs Col-V and Kα1T among healthy individuals is 2.9–5% [22, 23]. In our study, we identified a high prevalence of seropositivity among patients undergoing ARS (i.e., up to 69.8%), meaning that these patients may have unsuspected underlying parenchymal damage. Of note, in the present series, patients who presented with volume reflux (i.e., those who usually display a variety of typical GERD symptoms such as regurgitation and often atypical manifestations like cough, asthma, or dental erosions [24]) had a higher seroprevalence of abnormally elevated concentrations of SAbs than individuals who underwent ARS due to obstructive symptomatology (i.e., dysphagia) or complications resulting from a large HH (i.e., chronic anemia). Although inconclusive, this finding lends further evidence to the theory that aspiration of gastric contents may trigger the mechanism that induces the expression of SAbs.

There may be some caveats in using these humoral factors as a screening tool for lung damage among GERD patients. Indeed, the immunogenic profile of each SAg and the *de novo* expression of their corresponding SAb may differ. For example, factors including respiratory viral infections, smoking, and preexisting lung disorders may facilitate the initiation of this immune response, hence, the causes may be multifactorial [7]. In addition, a few studies have reported the abnormal expression of these SAbs in the context of other conditions, including breast cancer, small cell lung carcinoma, and transplant-related cardiomyopathy [25, 26].

This study has multiple limitations. There was a relatively small sample size, which limited further statistical analysis, and the single-center design may limit the generalizability of the findings. Also, our study lacks an objective respiratory evaluation (i.e., functional respiratory test or the use of validated tools) that may be relevant to establishing an association between SAb concentrations and clinical status or presentation. Furthermore, not all of the patients had complete preoperative testing, which precluded exploring correlations of SAb concentrations with specific pH metrics. In the future, it would be desirable to also include impedance testing to assess the correlation of SAbs with non-acidic reflux events as well as other potential diagnostic modalities (e.g., histological assessment, computed tomography, etc.) to document objective evidence of established lung damage. The use of other biomarkers in future research may help to determine whether aspiration is occurring (e.g., pepsin or pepsinogen A4 in bronchoalveolar lavage fluid or saliva). Finally, confounders such as autoimmune disorders or environmental factors were not assessed, and the study’s cross-sectional analysis and descriptive nature preclude causal inference.

## Conclusion

A high prevalence of abnormally elevated circulating humoral factors (i.e., anti-Col-V and/or anti-Kα1T) was found among patients with GERD and/or HH before ARS. This suggests that unsuspected extraesophageal complications of GERD, such as pulmonary damage (i.e., via silent micro-aspiration), are a relevant but often undetected problem. The high proportion of patients presenting with abnormal expression of SAbs was unexpected and raises serious concern for undiagnosed pulmonary parenchymal damage in this population, which should be further explored.

## Supplementary Information

Below is the link to the electronic supplementary material.

Supplementary material 1 (DOCX 32.3 kb)

## Data Availability

The data analyzed in this study is stored in a specific Research Electronic Data Capture system (RedCAP) that can be queried for future studies; however, it cannot be shared outside of those authorized as research staff per protocol. Please contact the corresponding author to access any data; an approval from the local IRB may be necessary.
